# Risk Factors for Drug Resistance in Epileptic Children with Age of Onset above Five Years: A Case-Control Study

**DOI:** 10.1155/2021/9092824

**Published:** 2021-11-10

**Authors:** Irawan Mangunatmadja, Raden Muhammad Indra, Dwi Putro Widodo, Achmad Rafli

**Affiliations:** ^1^Department of Child Health, Dr. Cipto Mangunkusumo Tertiary General Hospital-Faculty of Medicine Universitas Indonesia, Jakarta, Indonesia; ^2^Department of Child Health, Mohammad Hoesin General Hospital-Universitas Sriwijaya Medical School, Palembang, Indonesia

## Abstract

**Background:**

Children with epilepsy with onset above five years encompass distinct epidemiological and clinical characteristics that may have specific risk factors for resistance to antiseizure medications (ASMs). Studies on this age group are limited.

**Purpose:**

To identify risk factors for drug resistance in children with epilepsy with the age of onset above five years.

**Methods:**

A case-control study was conducted on children with epilepsy with the age of onset above five years visiting the Pediatric Neurology Clinic of Cipto Mangunkusumo and Mohammad Hoesin Hospital between September 2015 and August 2016. Cases consisted of drug-resistant children while control consisted of drug-responsive children according to 2010 ILAE classification. Risk factors studied include onset, number of seizures, illness duration before treatment, cause, seizure type, status epilepticus, initial and evolution of EEG, brain imaging, and initial treatment response.

**Results:**

Thirty-two pairs of children were included in the study. After logistic regression analysis, symptomatic etiology and failure to achieve early response to treatment were found to be associated with drug resistance with adjusted OR of 84.71 (95% CI: 5.18-1359.15) and 72.55 (95% CI: 7.08-743.85), respectively.

**Conclusion:**

Poor initial response to ASM and symptomatic etiology are independent risk factors for drug resistance in children with epilepsy with the age of onset above five years.

## 1. Introduction

Epilepsy is the most common neurological disorder, affecting 50 million people worldwide, with approximately 80% living in developing countries with limited resources. The incidence of epilepsy is the highest in childhood [1].

Antiseizure medications (ASMs) are still the mainstay treatment for epilepsy. However, around 10-40% of patients will have resistance to ASM [2–5], defined as the failure to achieve seizure control with two or more appropriate ASMs with adequate dose [6]. Drug resistance is the main problem complicating patients with epilepsy. Those who are resistant to ASMs have the highest mortality rate and risks for cognitive and behavioral problems. Children with epilepsy who are resistant to ASMs have a higher possibility for developmental disorders [7, 8].

Previous studies have shown that resistance to ASMs can be predicted early after diagnosis. Several clinical characteristics were found to be indicative of a higher risk for resistance to ASMs. Symptomatic etiology, history of perinatal insults, earlier age of onset, history of febrile seizure, presence of multiple types of seizure, complex partial seizure, abnormalities on EEG or brain imaging, and poor response to first drug have been associated with drug resistant epilepsy [4, 6, 9–15].

Children with epilepsy encompass a variety of age groups with different patterns of etiology, epidemiology, brain maturity, and clinical characteristics. Children with age of onset of epilepsy above five years may be a distinct group that may have different predictors of resistance to ASMs. In this age group, factors such as perinatal insults may cause less effect than younger children, and focal epilepsies may be more prevalent [4, 10, 16]. Several types of epilepsy syndromes are also associated with this age group that may be benign or resistant to medication, such as childhood or juvenile absence epilepsy, epilepsy with centrotemporal spikes, Panayiotopoulos syndrome, and juvenile myoclonic epilepsy [17, 18]. Previous studies indicate that different age groups may be differently affected by various known risk factors of drug resistance [4, 9, 16]. Studies regarding risk factors of ASMs resistance in children with epilepsy with an age of onset above five-year are lacking. Recent systematic reviews regarding risk factors of drug resistant epilepsy showed that no study specifically analyzed this age group and most studies analyzed children within a wide age group (0-18 years) or focused on younger children such as infants [4, 19]. Understanding risk factors of drug resistance in children with epilepsy with an age of onset above five-years is important because this group makes up a considerable amount of children with epilepsy, and more detailed knowledge will be useful to identify children at risk for drug resistance as early as possible to prepare appropriate measures.

## 2. Methods

This study was a case-control study on patients admitted between September 2015 and August 2016 to Cipto Mangunkusumo National Hospital Jakarta and Mohammad Hoesin Hospital. The inclusion criteria were children with onset of epilepsy above five years old counted from the first unprovoked seizure and had been treated with appropriately chosen and used ASMs [20]. The exclusion criteria were progressive illness such as Rett syndrome, tuberous sclerosis complex, or intracranial tumors. Patients with uncontrolled seizure and low compliance to treatment (concluded by interviewing the parents) were also excluded. Cases consisted of children with ASM resistance based on International League Against Epilepsy (ILAE) definition which is the failure to achieve sustained seizure freedom after administration of two or more appropriately chosen and used ASM schedules, while controls consisted of children who achieved sustained seizure freedom, defined as no seizure for at least 12 months or three times pretreatment interseizure interval (whichever is longer), using two or fewer ASM schedules [6]. Controls were matched for gender and age (less than two years of difference) to the case groups. The evaluation of diagnosis and treatment was done by pediatric neurology consultants.

Medical record review was performed to identify subjects. Patients were then examined and evaluated for their current drug responsiveness status and compliance. Written informed consent was obtained during these visits. Neurological examinations, reviews of initial EEG, and brain imaging results (if available) were done by the attending pediatric neurologist. To evaluate EEG evolution, a follow-up EEG was performed at the time of the study, thus separated by at least 12 months after the initial EEG. Both awake and sleep EEG recordings were taken. Risk factors studied included symptomatic etiology, age of onset, number of seizures before treatment, duration of illness before treatment, focal seizures, having more than one type of seizures, history of status epilepticus, abnormalities on brain imaging, abnormalities on initial EEG, the evolution of EEG, and initial response to treatment. Structural or symptomatic epilepsy was defined as the presence of neurological deficits on physical examination or abnormalities on brain imaging. Data were obtained from chart review and parents' or patients' interview during routine visits. Unfavorable EEG evolution is defined as abnormal follow-up EEG that includes epileptiform discharge, asymmetry, or background slowing. Unfavorable initial response was defined as the failure to achieve three consecutive months of seizure cessation within the first six months of ASM treatment [21].

Bivariate analysis performed using a *χ*^2^ test and multivariate analysis performed using logistic regression. Statistical Package for the Social Sciences (SPSS) version 18.0 software for Windows was used for analysis. The study protocol was reviewed and approved by the Ethical Committee for Health Studies of the University of Indonesia Medical Faculty.

## 3. Results

**Figure 1:**
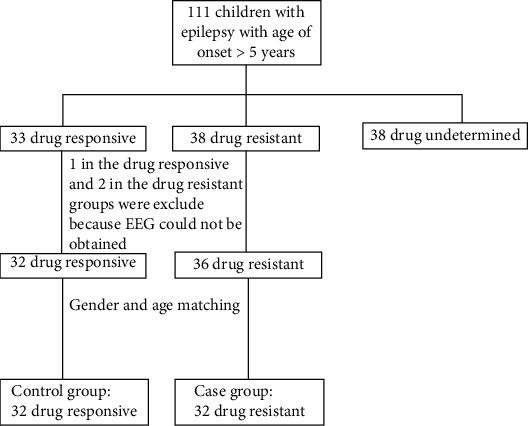
Flow diagram showing patients' selection.

During the study period, 111 eligible children were identified. Of these, 64 children were included in the study, 32 in each group (Figure 1). There were more girls than boys with a ratio of 1.9 to one. The characteristics of the subjects can be seen in Table 1. The number of drugs in the case group was significantly higher than controls. Valproic acid was the most common drug prescribed, which was to 29 and 23 children in control and case groups, respectively. The only other drug prescribed in the control group was phenytoin. Other drugs prescribed in the case group were phenytoin [22], carbamazepine [14], phenobarbital [11], levetiracetam [7], topiramate [6], and others [7]. Symptomatic epilepsy was identified in 26 children; most had hippocampal atrophy with or without sclerosis (7 children). Four children had cerebral atrophy and three with hemiatrophy without any sign of progression of the disease. The summary of symptomatic epilepsies is listed in Table 2. We identified several cases compatible with established epilepsy syndromes, all in the control group, including two cases with childhood absence epilepsy, two cases with epilepsy with generalized tonic clonic seizures only, one case with juvenile absence, one case with childhood epilepsy with centrotemporal spikes, and one with juvenile myoclonic epilepsy. History of perinatal asphyxia was recorded on three patients, one of which had neonatal seizures.

The results of the bivariate analysis are listed in Table 3. Factors identified to be significantly associated with ASM resistance were younger onset, symptomatic epilepsy, focal seizures, more than one type of seizure, abnormalities on brain imaging, unfavorable EEG evolution, and failure to achieve early seizure control. Brain imaging was only available on 44 patients, consisting of 31 MRIs and 13 CT scans. Brain imaging was typically prescribed for focal seizures or based on EEG characteristics of focal discharges or background slowing.

Multivariate analysis was performed using logistic regression including the following factors: the age of onset, etiological classification, number of seizures before treatment, focal seizure, multiple seizure types, history of status epilepticus, abnormalities on brain imaging, abnormal initial EEG, the evolution of EEG, and initial response to treatment. The results of the logistic regression analysis are listed in Table 4. Symptomatic etiology and unfavorable initial response to treatment were found to be independent risk factors with adjusted risks of 84.71 and 72.55, respectively.

## 4. Discussion

Our study was designed to identify risk factors for ASM resistance in children with epilepsy with the age of onset above 5 years. Data regarding this specific age group are still limited. This age group may not be exposed to risks for pathology found on perinatal or early infancy periods, such as genetic syndromes, birth asphyxia, and intracranial infections but still has a developing brain with increased susceptibility not found in young adults [23, 24]. The definitions of drug responsiveness and resistance used in this study were in accordance with the definition proposed by International League Against Epilepsy [6]. This may increase the quality of the results and facilitate comparison with other studies. The case-control design of this study may allow a stronger characterization of risk factors associated with ASM resistance.

We found a higher proportion of patients with younger onset, symptomatic epilepsy, focal seizure, presence of more than one type of seizure, abnormalities on brain imaging, unfavorable EEG evolution, and unfavorable initial response to treatment in the ASM resistant group compared to control. After logistic regression analysis, only symptomatic epilepsy and unfavorable initial response were independent risk factors for drug resistance.

Younger age of onset was more prevalent in the ASM resistant group. Previous studies had reported that the effect of age of onset was the strongest on those under one year old, even after controlling infantile spasm. However, onset up to age 9 years may still have negative effects on drug responsiveness, although the effect is diminishing with advancing age [25].

Focal seizures occurred in most of the subjects and were found in a higher proportion in the drug-resistant group. The presence of focal seizures has been known to be an independent risk factor for ASM resistance. [9] The higher proportion of focal seizures affecting both groups in this study might have caused the nonsignificant result in multivariate analysis. Patients with multiple types of seizures were not found in large numbers in this study, which also could contribute to the nonsignificant result. The finding of more than one type of seizure may indicate extensive or multiple epileptogenic foci [26, 27].

Unfavorable EEG evolution (changes in EEG findings at least 12 months after treatment) had a stronger effect on ASM resistance compared to initial EEG abnormalities. Previous studies on children age 0-18 years found a similar result [11, 13]; however, a study from the Dutch Study of Epilepsy found initial EEG to be a significant predictor of drug resistance [22]. The negative evolution of EEG might reflect increased the sensitivity of multiple examinations, but it might be the result of failure to control epileptic discharges due to nonresponse to treatment [28].

Two factors found independently associated with drug resistance were symptomatic epilepsy and unfavorable early response. Previous studies had also consistently found these factors to be associated with drug resistance across all age groups. The definition of initial response varies between studies, usually defined as seizure freedom within 3 to 12 months of the initial treatment. It is frequently found to be a strong predictor of long-term seizure freedom [11, 16, 29, 30]. In accordance with our own findings, other studies also found that children who had achieved seizures control did so on low to moderate ASM doses [31, 32].

As far as we know, there is no study on risk factors for drug-resistant epilepsy specifically in children with the age of onset above five years. Comparison to other studies which include children of different age groups might show some insight into the risk factors for drug resistance in our population. In a study that only included children with younger age of onset of younger than 36 months, risk factors for drug resistance that were found included the age of onset ≤ 12 months, abnormal brain imaging, developmental delay at diagnosis, and focal slowing on initial EEG after multivariate analysis [16]. The Nova Scotia Study and the Dutch Study of Epilepsy in Childhood, which included 1055 children with the age of onset of epilepsy between 1 month and 16 years, found symptomatic or cryptogenic epilepsy to be the most important risk factor after multivariate analysis [30, 33]. This result persisted after 15 years of follow-up [34]. Other studies exclusively in children also found younger age of onset (between <1 and 6 years), multiple seizure types, and abnormal EEG to be independent risk factors for drug resistance after multivariate analysis [11–16], in which our study did not demonstrate. A large number of studies also included a combined analysis of adult and pediatric populations. A recent meta-analysis that included 38 studies and 13.080 subjects similarly found symptomatic etiology, age of onset, abnormal brain imaging, status epilepticus, mental retardation, neurologic abnormality, psychiatric comorbidity, abnormal EEG, and febrile seizure to be risk factors for drug-resistant epilepsy [4], but did not find the initial response to treatment and focal seizure to increase the risk of intractability as in aforementioned studies which exclusively recruited children. The 2017 ILAE new classification of epilepsy [35] divided previously symptomatic epilepsy into structural, infectious, metabolic, or immune etiologies. Because of the exclusion of progressive illness in this study, symptomatic cause would be limited to structural epilepsy.

The small sample size in this study may be the limiting factor that prevented the true characterization of the degree of risk each factor presented in this study. Very high odds ratios found suggested heterogeneity between compared groups but can also be caused by the small sample size, a weakness of this study. Future studies should probably separate between symptomatic and genetic epilepsies and incorporate a larger sample size.

Another weakness of this study is that not all patients had brain imaging, specifically MRI. Brain imaging in epilepsy is not recommended on all patients. Most brain imaging in this study was performed on the symptomatic or drug-resistant group with a high yield of abnormalities, although after adjustment it was not found to be an independent risk factor, unlike symptomatic etiology in our study. This may be due to the smaller number of subjects who had brain imaging. Performing brain imaging on all subjects may allow better characterization of its result as a risk factor for drug resistance. Serum drug level examinations also were not available in this study, thus not allowing the analysis of the association between pharmacodynamics of the drugs with their effects. Data regarding cognitive function was not available in this study. Such data might be useful to better characterize patients' condition. Finally, it has been documented that even though survivors of pre- or perinatal brain injury usually will have early onset epilepsy, some conditions such as perinatal arterial ischaemic stroke can have a long latent period of over 5 years [36]. Detailed data regarding perinatal illness were not available in our study.

In conclusion, this study found symptomatic epilepsy and unfavorable initial response (failure to achieve three consecutive months of seizure freedom within the first six months of treatment) to be independent risk factors for resistance toward ASMs in children with epilepsy with the age of onset above five years. These risk factors should be identified early in the course of the treatment so that special precautions can be made. Dose escalation should be done rapidly, and parents should receive counseling for possible prognosis, polytherapy, and other treatment modalities.

## Figures and Tables

**Table 1:** Characteristics of subjects in the case and control groups.

Characteristics	Case	Control	Total
Sex			
Male	11 (34.4%)	11 (34.4%)	22 (34.4%)
Female	21 (65.6%)	21 (65.6%)	42 (65.6%)
Age (mean [SD] in months)	143.72 (35.21)	145.48 (32.87%)	
Age groups (*n*)			
>5–9 years	6 (18.8%)	6 (18.8%)	12 (18.8%)
>9–14 years	18 (56.2%)	18 (56.2%)	36 (56.2%)
>14 years	8 (25.0%)	8 (25.0%)	16 (25.0%)
Age of onset (mean [SD] in months)	92.19 (25.70)	104.41 (31.51)	
Average number of drugs used^∗^	3.19	1.09	
Monotherapy	30 (93.75%)	0 (0%)	30 (46.87%)
Polytherapy	2 (6.25%)	32 (100%)	34 (53.13%)

^∗^All comparisons between both groups were not statistically significant (*p* > 0.05) except for the number of drugs used (*p* < 0.001).

**Table 2:** Description of 26 patients with symptomatic epilepsies.

Description of symptomatic epilepsies	Case	Control	*N*
Hippocampal atrophy with or without sclerosis	7	0	7
History of encephalitis or bacterial meningitis	4	0	4
Cerebral infarct/atrophy	4	0	4
Cerebral palsy	2	1	3
Vascular malformation	2	0	2
Arachnoid cyst	2	0	2
Schizencephaly	1	0	1
Tuberculoma	0	1	1
Cerebral hemorrhage	1	0	1
Cerebral toxoplasmosis	0	1	1

**Table 3:** Bivariate analysis of factors associated with AED resistance.

Variables	Case	Controls	*p* ^∗^	OR	95% CI
Age of onset (year)					
>5–9	26	18	0.031	3.37	1.09–10.43
>9	6	14			
Etiology					
Symptomatic	23	3	<0.001	24.70	5.99–101.86
Idiopathic	9	29			
Pretreatment duration of illness (year)					
>1	6	5	0.740	1.25	0.34–4.59
≤1	26	27			
Pretreatment number of seizures					
>10 times	15	10	0.200	1.94	0.70–5.38
≤10 times	17	22			
Focal seizure					
Yes	25	14	0.005	4.59	1.54–13.67
No	7	18			
Multiple seizure types					
Yes	11	4	0.039	3.67	1.02–13.14
No	21	28			
History of status epilepticus					
Yes	4	9	0.120	2.74	0.75–10.06
No	28	23			
History of febrile seizure					
Yes	12	9	0.298	1.53	0.54–4.39
No	20	23			
Brain imaging (*n* = 44)					
Abnormal	21	3	<0.001	13.0	2.85–59.39
Normal	7	13			
Initial EEG					
Abnormal	22	15	0.076	2.49	0.90–6.91
Normal	10	17			
EEG evolution					
Not favorable	18	5	0.01	6.94	2.13–22.65
Favorable	14	27			
Initial response to treatment					
Not favorable	30	6	<0.001	65.0	12.06–350.25
Favorable	2	26			

Chi square test.

**Table 4:** Results of logistic regression analysis for risk factors of AED resistance.

Variable	p	OR	95% CI
Pretreatment seizures > 10 times	0.050	9.59	0.99-90.58
Symptomatic epilepsy	0.002	82.29	5.12-1323.27
Unfavorable initial response to treatment	<0.001	70.99	6.94-726.23

## Data Availability

Data and material are available for transparency.

## Additional Points

*Code Availability*. Program Software SPSS vers.20.
